# Myelodysplastic Syndrome With Complex Chromosomal Karyotype Abnormalities Complicated by Multiple Intestinal Perforations: A Case Report and Literature Review

**DOI:** 10.1155/crgm/2593347

**Published:** 2026-01-08

**Authors:** Zhan-Yue Niu, Ming-Fei Zhu, Ming Tao, Cheng Zhang, Fang Gu, Jun Li

**Affiliations:** ^1^ Department of Gastroenterology, Peking University Third Hospital, No. 49 Huayuan North Road Haidian District, Beijing, China, puh3.net.cn; ^2^ Department of General Surgery, Peking University Third Hospital, No. 49 Huayuan North Road Haidian District, Beijing, China, puh3.net.cn

**Keywords:** Behçet-like disease, complex chromosomal karyotype, myelodysplastic syndrome (MDS), small bowel perforation, trisomy 8

## Abstract

**Background:**

Myelodysplastic syndromes (MDSs) are clonal hematopoietic disorders often associated with cytogenetic abnormalities, among which trisomy 8 is one of the most common abnormalities. Trisomy 8 is linked to autoimmune manifestations, including Behçet‐like disease, especially with gastrointestinal involvement.

**Case Presentation:**

We report a rare and severe case of a 59‐year‐old male with MDS characterized by a complex abnormal karyotype, including trisomy 8, accompanied by multiple ileal perforations and intestinal ulcers resembling Behçet’s disease. Despite surgical intervention and broad‐spectrum antimicrobials, recurrent symptoms persisted. A combination of corticosteroids and thalidomide ultimately led to clinical and hematologic improvement.

**Methods:**

Clinical, radiologic, histopathologic, and cytogenetic data were collected. Literature review was conducted to contextualize diagnostic criteria and treatment strategies.

**Results:**

The patient showed hematologic and symptomatic improvement following immunosuppressive therapy. Chromosome karyotype analysis revealed chromosomal numerical abnormalities, including trisomy of chromosomes 8, 9, and 15 and the presence of an extra Y chromosome. Gastrointestinal involvement with recurrent perforations was attributed to Behçet‐like intestinal disease.

**Conclusion:**

This case underscores the aggressive clinical course and diagnostic challenges of Behçet‐like disease associated with MDS and trisomy 8, highlighting the importance of early recognition and immune‐targeted therapy.

## 1. Introduction

Myelodysplastic syndromes (MDSs) are a heterogeneous group of clonal hematopoietic stem cell disorders characterized by ineffective hematopoiesis, peripheral cytopenias, and an increased risk of progression to acute myeloid leukemia. Among the cytogenetic abnormalities associated with MDS, trisomy 8 is one of the most frequently identified abnormalities and is notably linked to a spectrum of immune‐mediated complications, including Behçet‐like disease [1]. Behçet‐like manifestations in MDS, particularly those involving the gastrointestinal tract, present a unique clinical challenge due to their aggressive course, high risk of intestinal perforation, and limited therapeutic options.

While classic Behçet’s disease is defined by recurrent oral and genital ulcers, uveitis, and systemic vasculitis, its intestinal variant—particularly when associated with trisomy 8 MDS—often lacks full systemic features and presents with deep, refractory ulcers primarily involving the ileocecal region. The pathogenesis of this overlap syndrome remains incompletely understood, though aberrant immune activation, potentially driven by chromosome 8 gene dysregulation, is implicated [2].

We present a rare and severe case of MDS characterized by a complex abnormal karyotype, including trisomy 8, complicated by multiple small bowel perforations and Behçet‐like intestinal ulcers. Through a comprehensive clinical, radiologic, histopathologic, and cytogenetic evaluation, this report underscores the importance of early recognition and multidisciplinary management of this high‐risk entity. A review of the current literature is also provided to contextualize diagnostic and therapeutic strategies in this complex overlap syndrome.

## 2. Case Presentation

### 2.1. Baseline

A 59‐year‐old man was admitted with a 2‐month history of abdominal pain and over 1 month of intermittent fever. On 13 March 2025, the patient developed periumbilical cramping pain that resolved spontaneously and was accompanied by oral ulcers. On 26 March, gastroscopy showed chronic nonatrophic gastritis, and colonoscopy revealed multiple colonic polyps treated with argon plasma coagulation. On 29 March, he developed fever up to 40.0°C with persistent worsening abdominal pain (VAS 6/10). On 11 April, laboratory tests revealed severe pancytopenia (WBC 2.82 × 10^9^/L, Hb 105 g/L, MCV 103 fL, RDW‐SD 73.10 fL, RDW‐CV 17.90%, and PLT 79 × 10^9^/L), and abdominal CT demonstrated free intraperitoneal air and peritonitis. He underwent emergent surgery for ileal perforation repair and peritoneal lavage. Despite surgery, fever and pain persisted. On 29 April, labs showed further pancytopenia (WBC 0.64 × 10^9^/L, Hb 57 g/L, MCV 110 fL, RDW‐SD 71 fL, RDW‐CV 18%, and PLT 40 × 10^9^/L). CT suggested intestinal obstruction with bowel wall thickening and dilatation. Empiric treatment with imipenem–cilastatin and vancomycin, plus total parenteral nutrition, led to transient symptomatic relief. Attempts to reintroduce enteral feeding consistently triggered pain, necessitating continued parenteral support. On 13 May, he was transferred to the gastroenterology department of Peking University Third Hospital. The patient had no prior medical history and unremarkable personal, reproductive, or family history.

Physical examination on admission (13 May) revealed the following: body temperature 36.5°C, heart rate 86 beats/min, respiratory rate 17 breaths/min, and blood pressure 119/78 mmHg. The patient was conscious. Lung and cardiac examinations were unremarkable. The abdominal wall showed a nonhealed surgical incision that was firm on palpation. There was tenderness with rebound tenderness in the right abdomen, without guarding. Bowel sounds were decreased (2 times/min). No edema was observed in the lower limbs. Relevant laboratory findings during hospitalization are summarized in Table 1.

**Table 1:** Inpatient laboratory and imaging investigations.

Test	Result	Reference/interpretation
White blood cell count	1.17 × 10^9^/L	Decreased
Absolute neutrophil count	0.69 × 10^9^/L	Decreased
Red blood cell count	2.82 × 10^12^/L	Decreased
Hemoglobin	87 g/L	Decreased
Mean corpuscular volume	96.10 fL	Normal
Red blood cell distribution width	63.80 fL	Elevated
Red blood cell distribution width‐CV	18.40%	Elevated
Platelet count	33 × 10^9^/L	Decreased
C‐reactive protein (CRP)	104.4 mg/L	Elevated
Erythrocyte sedimentation rate (ESR)	24 mm/h	Mildly elevated
Procalcitonin	0.25 ng/mL	Slightly elevated
Fecal calprotectin	347.08 μg/g	Elevated
Albumin	23.6 g/L	Decreased
Renal function	Normal	—
Serum potassium	3.16 mmol/L	Hypokalemia
Serum iron	6.81 μmol/L	Decreased
Total iron‐binding capacity	20 μmol/L	Normal
Ferritin	1369 ng/mL	Elevated (likely inflammation‐related)
Folate	8.1	Normal
Reticulocyte percentage	0.45%	Decreased
Prothrombin time	15.2 s	Prolonged
Prothrombin activity	62%	Decreased
International normalized ratio (INR)	1.43	Mildly elevated
D‐dimer	1.46 μg/mL	Elevated
CMV IgM/DNA	Negative	—
EBV IgM/DNA	Negative	—
G test/GM test	Negative	—
Fecal culture, fungi, *Mycobacterium tuberculosis*, parasites, *C. difficile*	Negative	—
T‐SPOT.TB (IFN‐γ release assay)	Negative	—
ANA, ANCA, anti‐dsDNA antibodies	Negative	—
Tumor markers	Within normal limits	—

**Figure 2:**
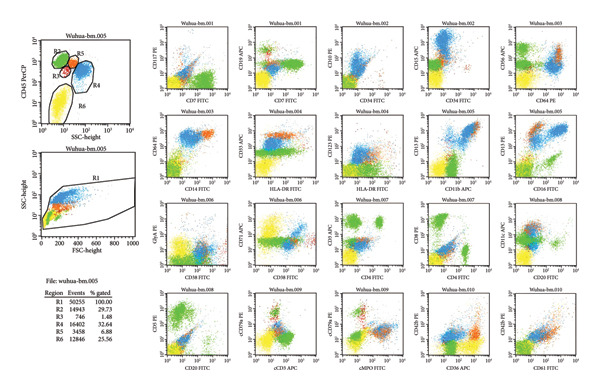
Bone marrow immunophenotyping by flow cytometry. Flow cytometric analysis of the bone marrow aspirate demonstrates a decreased proportion of mature granulocytes with evidence of dysgranulopoiesis. Aberrant expression of CD56 is observed in a subset of mature granulocytes and monocytes.

**Figure 3:**
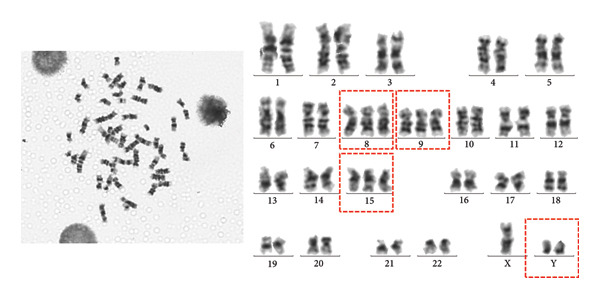
Conventional cytogenetic analysis of bone marrow cells. G‐banded karyotype analysis was performed on 20 metaphase cells. Nine cells exhibited a hyperdiploid karyotype with numerical chromosomal abnormalities, including gain of the Y chromosome and trisomy of chromosomes 8, 9, and 15, consistent with acquired cytogenetic abnormalities.

Abdominal CT revealed ascites, peritonitis, and small‐bowel changes. The bone marrow cytological examination revealed that the proliferation of nucleated cells was extremely low, with the granulocyte component accounting for 42%. Some granulocytes showed toxic changes; the erythroid component accounted for 20%, with varying sizes of mature red blood cells; lymphocytes, monocytes, and plasma cells were all present, but megakaryocytes were absent, and platelets were rare; 20% of ring sideroblasts were observed (Figure 1). The immunophenotyping showed that the proportion of mature granulocytes was decreased, with abnormal granulocyte development, and some mature granulocytes and monocytes expressed CD56, while the proportion of early cells was reduced. The above findings are consistent with MDS (Figure 2). FISH analysis of 200 interphase cells revealed that trisomy of chromosome 8, associated with the CEP8 gene, was present in 54% of the cells. The results of the chromosomal karyotype analysis revealed that out of 20 metaphase cells examined, 9 exhibited a hyperdiploid karyotype with chromosomal numerical abnormalities, including trisomy of chromosomes 8, 9, and 15 and the presence of an extra Y chromosome, which are acquired cytogenetic abnormalities (Figure 3). The pathergy test was negative. Ophthalmologic exam found no Behçet‐related signs.

Figure 1Morphological evidence supporting the diagnosis of MDS. (a) Bone marrow cytology shows markedly reduced nucleated cell proliferation, anisopoikilocytosis of mature erythroid cells, and absence of megakaryocytes. (b) Iron staining demonstrates the presence of ring sideroblasts.

**Figure:**
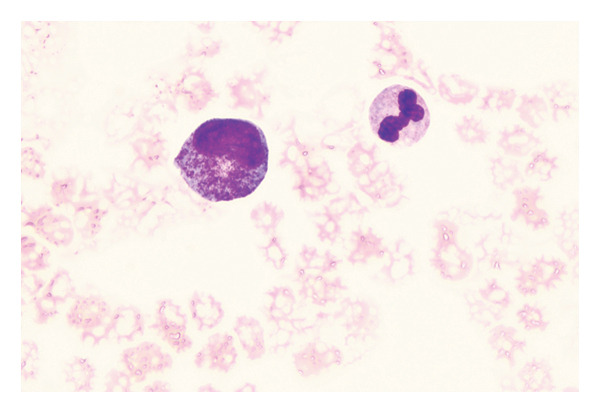
(a)

**Figure:**
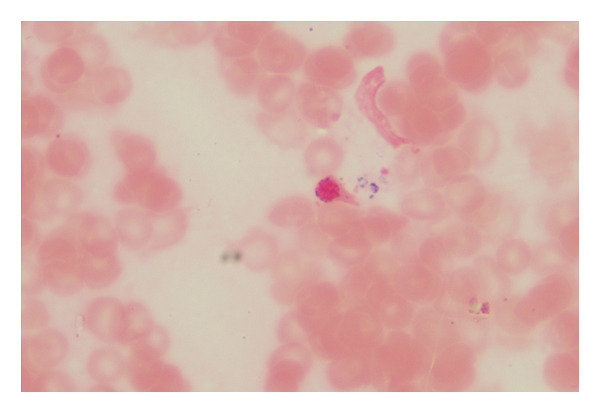
(b)

### 2.2. Clinical Course and Management

The patient received imipenem–cilastatin and vancomycin, total parenteral nutrition, albumin and potassium correction, G‐CSF for leukopenia, and hetrombopag for thrombocytopenia. Wound care was continued. On 15 May, he developed low‐grade fever (37.5°C) with VAS 4–5 abdominal pain. Blood cultures were negative; pathogen sequencing yielded suspected human herpesvirus 4. Vancomycin trough levels were subtherapeutic and were subsequently adjusted, but fever persisted. On 21 May, hypotension (BP 78/48 mmHg), bradycardia (HR 37 bpm), and arrhythmia occurred, with worsening abdominal pain. ECG demonstrated sinus bradycardia with irregular rhythm, but no ST‐T changes. Suspected septic shock triggered intravenous fluids, norepinephrine and dopamine infusion, and placement of a temporary pacemaker. He was transferred to the ICU and underwent emergency laparotomy, which revealed over 10 ileal perforations and severe adhesions (Figure 4). Roughly 1 m of small intestine was resected, and a single‐barrel ileostomy was fashioned. Postoperative peritoneal fluid culture grew *Pseudomonas aeruginosa*, resistant to imipenem, meropenem, piperacillin/tazobactam, ceftazidime, ciprofloxacin, and levofloxacin, but sensitive to amikacin and ceftazidime/avibactam. Antibiotics were switched accordingly, and fever resolved. Surgical wound required debridement and dressing changes, followed by gradual healing. Pathology revealed ulceration and perforation with mucosal inflammation and subserosal abscesses; occasional multinucleated giant cells; and no vasculitis. Immunohistochemistry was CMV‐negative; EBER in situ hybridization negative; and PAS/PASM negative. Diagnosis: Myelodysplastic syndrome with a complex abnormal karyotype, complicated by Behçet‐like intestinal involvement. Based on the WHO (2016) revised classification for MDS, the diagnosis is MDS with ring sideroblasts and multilineage dysplasia (MDS‐RS‐MLD).

Figure 4Gross examination of surgical specimens. (a) Resected small intestinal specimen showing multiple segments of involvement; the yellow circle highlights a visible perforation. (b) Magnified view of the affected segment with a clearly demarcated perforation indicated by the yellow arrow.

**Figure:**
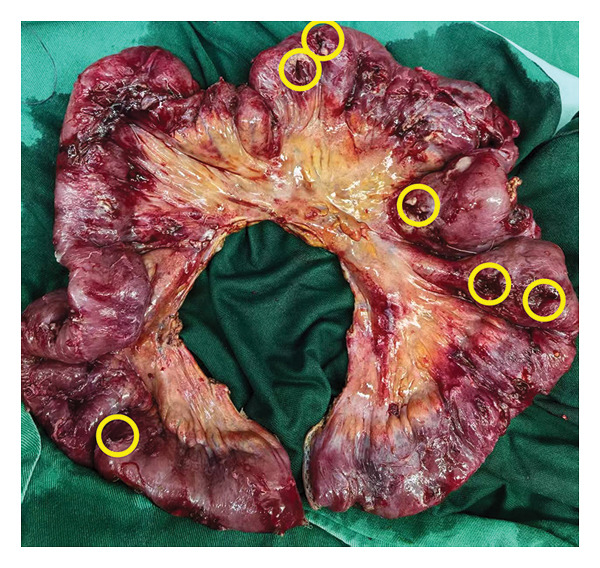
(a)

**Figure:**
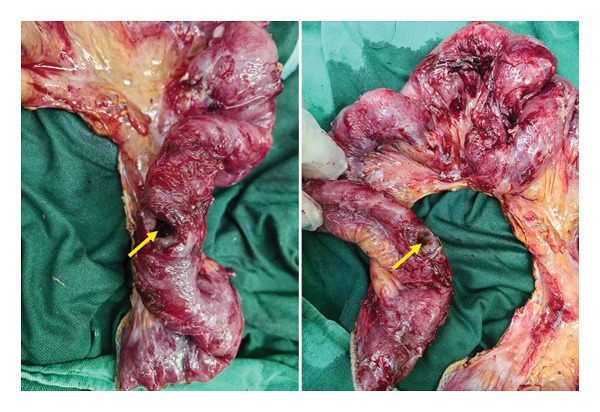
(b)

Post‐ileostomy, multiple new small intestinal ulcers (0.3–0.5 cm) were noted. Intravenous methylprednisolone 40 mg/day was initiated. After 5 days, ulcers healed; enteral nutrition was restarted. Methylprednisolone tapering continued, and thalidomide was added (starting 25 mg/day escalating to 100 mg/day). Fever and abdominal pain have resolved. Enteral nutrition and oral intake are gradually advancing. Latest labs show WBC 1.77 × 10^9^/L, Hb 102 g/L, MCV 99.1 fL, RDW‐SD 82 fL, RDW‐CV 23%, and PLT 138 × 10^9^/L.

## 3. Discussion

MDS represents a heterogeneous clonal hematopoietic stem‐cell disorder characterized by chronic cytopenias with dysplastic traits and carries a high risk for progression to acute myeloid leukemia [3]. Approximately 10%–20% of MDS patients exhibit concomitant autoimmune manifestations such as systemic vasculitis or connective tissue diseases [4, 5]. Notably, an association between MDS and Behçet‐like disease has been described, often featuring trisomy 8 and gastrointestinal ulcerations [6]. Among MDS patients with Behçet‐like features, trisomy 8 is identified in 81.3%, and among trisomy 8 MDS cases, 76.9% have Behçet‐like symptoms, with 69.2% presenting intestinal involvement [7].

Mechanistically, trisomy 8 may lead to overexpression of surface antigens, triggering autoreactive T‐cell proliferation and resultant marrow failure and autoimmunity [8]. The MYC oncogene (on chromosome 8) and other immunoregulatory genes on chromosome 8 may contribute to aberrant epithelial regeneration, immune dysregulation, and cytokine‐driven intestinal ulcer formation.

Patients with MDS and Behçet‐like disease average 42.6 years old, with female predominance and younger onset in women. In two‐thirds, intestinal lesions precede MDS diagnosis; nearly half present simultaneously; few occur after MDS onset. Common symptoms include abdominal pain and fever; ocular involvement is uncommon. Gastrointestinal ulcerations typically affect the ileum, ileocecal region, colon, and rectum and can lead to deep ulcerations, perforation, and bleeding [9–15].

Diagnostic criteria for gastrointestinal Behçet‐like disease differ from systemic Behçet’s disease; histologic vasculitis is infrequent and not required. The pathergy test has high specificity (98.4%) but low sensitivity (35.8%) [16]. Only 14.3% meet full systemic Behçet’s criteria [16]. The Korean group’s criteria include oral ulceration and atypical intestinal lesions, supporting a presumptive diagnosis even when Crohn’s disease is ruled out. Markedly elevated fecal calprotectin, as in this case, correlates with disease severity [17].

This patient initially presented with abdominal pain and fever and was subsequently found to have multiple ileal ulcers complicated by recurrent intestinal perforations. While intestinal perforation has been reported in similar cases, it remains relatively uncommon. In this case, extensive intra‐abdominal adhesions may have masked classic signs of peritonitis, complicating timely clinical assessment and diagnosis.

According to the diagnostic criteria proposed by the Korean Association for the Study of Intestinal Behçet’s Disease, the presence of oral ulcers along with atypical intestinal ulceration supports a presumptive diagnosis of Behçet’s disease [18]. Although fecal calprotectin was markedly elevated, the patient did not meet diagnostic criteria for Crohn’s disease. Prior studies have shown that intestinal Behçet’s disease can lead to significant elevations in fecal calprotectin, which also correlates with disease severity.

Given the presence of MDS with chromosome 8 trisomy, the diagnosis of trisomy 8–associated MDS with Behçet‐like intestinal manifestations was established. Current research on the treatment of this condition remains limited [8, 19]. Allogeneic hematopoietic stem cell transplantation may be the most effective therapeutic approach. Glucocorticoids have demonstrated an approximate response rate of 53%, while other agents such as thalidomide, cytarabine, decitabine, and cyclosporine have also shown efficacy [8]. However, more than half of patients require long‐term corticosteroid maintenance, and relapses are common [20]. 5‐Aminosalicylic acid and tumor necrosis factor‐α (TNF‐α) inhibitors may offer some benefit for intestinal Behçet‐like lesions, though the therapeutic effect is generally inferior to MDS‐directed therapies. Isolated surgical management has been reported in select cases to result in durable remission, though this appears to be the exception [21]. Thalidomide has been used in the treatment of MDS, as reported in previous studies [22–24], and has also shown efficacy in some patients with MDS complicated by intestinal Behçet’s disease [8]. The mechanisms by which thalidomide exerts therapeutic effects in MDS are multifaceted. It can reduce apoptosis levels [25] and modulate the bone marrow microenvironment by promoting IL‐10 expression in bone marrow stromal cells, thereby effectively delaying disease progression [26]. In addition, the direct molecular target of thalidomide, cereblon (CRBN), serves as the substrate‐recognition subunit of the CRL4 ubiquitin ligase complex, which is responsible for identifying and mediating the ubiquitination and degradation of specific proteins. By binding to CRBN, thalidomide induces conformational changes that alter substrate specificity, leading to the ubiquitination and degradation of IKZF1 and IKZF3, thereby suppressing malignant hematopoietic cell proliferation. Its derivative lenalidomide further promotes the degradation of casein kinase 1α (CK1α), activates the p53 pathway, and induces apoptosis in tumor cells [27].

In the present case, new intestinal ulcers emerged following surgery, suggesting that surgical intervention alone did not achieve full disease control. Subsequent administration of corticosteroids led to ulcer healing, and the addition of thalidomide was associated with recovery of leukocyte, hemoglobin, and platelet counts, indicating treatment efficacy. The favorable therapeutic outcome observed in our patient further supports thalidomide as an effective treatment option for MDS complicated by intestinal Behçet‐like disease.

In hematologic outcomes (leukemic transformation and life‐threatening cytopenias), prognosis is similar to isolated MDS. However, gastrointestinal complications, especially perforations, carry significant mortality risk [12]. Given the nonspecific presentation and high risk of delayed diagnosis, clinicians should consider this diagnosis in patients with abdominal pain, fever, and cytopenias. Large‐scale studies are needed to define optimal, long‐term treatment regimens.

## 4. Conclusion

This case illustrates the aggressive nature of Behçet‐like intestinal disease in MDS with trisomy 8. Early diagnosis and immune‐targeted therapy are crucial. Surgical treatment alone may be insufficient. Corticosteroids and thalidomide appear effective in inducing remission. Increased clinical awareness is essential to improve outcomes.

## Consent

Written informed consent was obtained from the patient for publication of this case report and associated images.

## Disclosure

All authors reviewed and approved the final manuscript.

## Conflicts of Interest

The authors declare no conflicts of interest.

## Author Contributions

Zhan‐Yue Niu was responsible for the conception, data collection, and drafting of the manuscript. Ming‐Fei Zhu, Ming Tao, and Cheng Zhang assisted in clinical data acquisition and imaging interpretation. Fang Gu contributed to pathological evaluation and literature review. Jun Li supervised the study, revised the manuscript critically for important intellectual content, and is the corresponding author.

## Funding

This study was funded by the China Health and Medical Development Foundation (LM2023782), the International Institute of Population Health, Peking University Health Science Center (JKCJ202305), and the Clinical Cohort Development Project of Peking University Third Hospital (BYSYDL2025009).

## Data Availability

Data sharing is not applicable to this article as no datasets were generated or analyzed during the current study.
